# Molecular Detection of Equine Adenovirus 1 in Nasal Swabs from Horses in the Republic of Korea

**DOI:** 10.3390/vetsci9040187

**Published:** 2022-04-13

**Authors:** Sang-Kyu Lee, Jeechan Choi, Jungho Yoon, Jaemin Jung, Joon-Young Park, Jongyoung Park, Yeonjong Kim, Ji-Young Park, Dongsun Park

**Affiliations:** 1 Veterinary Center, Korea Racing Authority, Gwacheon 13822, Korea; bestvet@kra.co.kr (S.-K.L.); dc3412@kra.co.kr (Y.K.); 2 Busan Equine Hospital, Korea Racing Authority, Busan 46745, Korea; jeechan9202@kra.co.kr; 3 Equine Clinic, Jeju Regional Headquarter, Korea Racing Authority, Jeju 63346, Korea; junghoy11@gmail.com (J.Y.); jaemin@kra.co.kr (J.J.); penditis@gmail.com (J.P.); 4 Jangsu Equine Hospital, Korea Racing Authority, Jangsu 55620, Korea; vetzune@kra.co.kr; 5 Animal Disease Diagnostic Division, Animal and Plant Quarantine Agency, Gimcheon 39660, Korea; jijipy@korea.kr; 6 Department of Biology Education, Korea National University of Education, Cheongju 28173, Korea

**Keywords:** EAdV-1, prevalence, nasal swabs, phylogenetic analysis, Korea

## Abstract

Equine adenovirus 1 (EAdV-1) can cause upper respiratory disease in horses and has been reported worldwide. In this study, and for the first time in Korea, the prevalence of EAdV-1 in equine nasal swabs was investigated using a PCR to identify potential risk factors and examine the genetic diversity of its DNA sequences by a comparison with foreign strains. Nasal swabs collected from 359 horses reared at Korea Racing Authority facilities were tested using an EAdV-1 hexon-specific PCR and the associations between EAdV-1 infection and sex, age, region, breed, and activity were analyzed. Five samples (1.4%, 5/359) tested positive for EAdV-1; however, no statistically significant differences were observed with respect to any variable. Among the five EAdV-1-positive horses, a co-infection with equine influenza, equine herpesvirus 1 and 4, or *Streptococcus equi* was not detected; however, clinical respiratory signs were observed in one. Phylogenetic analyses based on partial EAdV-1 hexon gene sequences revealed that the Korean EAdV-1 isolates shared approximately 98.8–100% similarity among each other and with foreign strains. Three Korean isolates shared high similarity with strains from Australia and India and the remaining two isolates were separate in phylogenetic analyses. These findings highlight the molecular prevalence and genetic diversity of EAdV-1 in horses in Korea.

## 1. Introduction

Viral respiratory disease is an important cause of morbidity in horses and it may hinder training and racing programs in performance horses [1,2]. Even mild respiratory diseases can hinder peak performance in horses [3]. Many viral infections have been associated with respiratory diseases in horses [1]. A survey conducted among equine veterinarians indicated that viral respiratory disease was the second most important equine medical problem [4].

Most adenoviruses are host-specific; they are isolated from the upper respiratory tract in animals and cause subclinical infections, particularly upper respiratory diseases [5]. Two equine adenoviruses have been reported, equine adenovirus 1 (EAdV-1) and equine adenovirus 2 (EAdV-2) [5]. EAdV-1 is primarily associated with the upper respiratory tract whereas EAdV-2 affects the gastrointestinal tract [6].

Although EAdV-1 has been isolated from both clinically affected and healthy horses [7,8,9], it is a common and important cause of virus-induced respiratory disease in horses [10]. An EAdV-1 infection is subclinical or associated with mild upper respiratory tract diseases [5,8,10]. The clinical signs of an EAdV-1 infection in equine upper respiratory disease include nasal discharge, coughing, an enlarged submandibular lymph node, and pyrexia [8,11]. In Arabian and thoroughbred foals, EAdV-1 infections have been reported to result in severe and occasionally fatal pneumonia [12]. EAdV-1 is ubiquitous in horses and readily isolated from both healthy horses and horses with clinical signs [7,11]. Since a 24.1% EAdV-1 seroprevalence was first reported in British horses [13], several serological studies have been conducted in various countries with reported seroprevalence findings of 82.7% and 8.6% in Japan [14,15], 54.9% and 77% in Australia [6,16], 39.9% and 91.3%% in New Zealand [17,18], 10.6% in Ireland [19], 4.5% in Nigeria [20], and 39.0% in the Netherlands [21]. A polymerase chain reaction (PCR) assay is an important method in EAdV-1 diagnosis, and PCRs targeting the EAdV-1 hexon gene have been commonly used in previous studies [10,12,22,23,24]. However, relatively limited studies have been conducted on the molecular detection of EAdV-1 from nasal swabs, compared with seroprevalence studies. To date, five EAdV-1 detections using a PCR assay of equine nasal swabs have been reported from Australia, the United States, Turkey, France, and Belgium [22,24,25,26,27].

The Korea Racing Authority (KRA) is the only horseracing company in the Republic of Korea; approximately 3300 horses are reared at KRA facilities in four regions. A previous study reported that 29.6% of horses at a KRA facility had a yearly infectious respiratory disease [28]. Infectious respiratory viruses, including equine herpesvirus type 1 (EHV-1), equine herpesvirus type 4 (EHV-4), equine herpesvirus type 5 (EHV-5), equine rhinitis A virus (ERAV), and equine rhinitis B virus (ERBV), have been investigated using nasal swab samples from horses at the KRA facility in Seoul [29]. However, EAdV-1 detection from nasal swab samples has not yet been conducted in the Republic of Korea. Therefore, this study investigated and genetically characterized the Korean strains of an EAdV-1 infection from the nasal swabs of horses reared at KRA facilities in the Republic of Korea.

## 2. Materials and Methods

### 2.1. Samples

This study included 359 horses (112 males, 123 geldings, and 124 females) aged 2–23 years with a mean age of 5.9 years from KRA branch facilities in four regions in the Republic of Korea (Table 1). Nasal swab samples were collected according to the Institutional Animal Care and Use Committee of the Korea Racing Authority guidelines (Reference number: AEC-2101). The clinical signs of respiratory disease were recorded at the sample collection. Horses with a cough or nasal discharge were considered to be affected by respiratory diseases.

### 2.2. PCR Detection of EAdV-1 and Genetic Characterization of the Hexon Gene

Viral DNA was extracted from nasal swab samples using a Maxwell^®^ RSC Viral Total Nucleic Acid Purification Kit (Promega, Madison, WI, USA) on a Maxwell^®^ RSC 48 Instrument (Promega) according to the manufacturer’s instructions and stored at −80 °C. For EAdV-1 DNA screening, the PCR was performed using oligonucleotide primers targeting a 299 bp-specific region of the EAdV-1 hexon gene, as previously described [10,23]. The PCR products were visualized on 1.5% tris–acetate–EDTA agarose gel using Safe Shine Green (Biosesang, Seongnam, Korea) under a UV light. The results were confirmed by a sequence analysis of the Korean strains. The amplified DNA products were purified using the PureLink™ Quick Gel Extraction and PCR Purification Combo Kit (Invitrogen™, Waltham, MA, USA) and sequenced using an ABI PRISM BigDye Terminator Cycle Sequencing Ready Reaction Kit V.3.1 (Applied Biosystems, Waltham, MA, USA) with an ABI3730 Genetic Analyzer (Applied Biosystems). After sequencing the amplicons using the same primers, the sequences from both directions were assembled and the homology of the deduced nucleotide sequences for EAdV-1 was analyzed using the GenBank (http://www.ncbi.nlm.nih.gov/Genbank/, accessed on 1 March 2022) databases.

Phylogenetic analyses were used to determine the genetic relationships between the Korean EAdV-1 and the isolates from other countries. The three nucleotide sequences of the EAdV-1 hexon gene obtained from GenBank were used for the analysis. The EAdV-1 hexon gene sequences identified in this study were aligned with those retrieved from GenBank using BioEdit v.7.2.6 and MEGA 11 using the Clustal W algorithm. The sequences were trimmed to the partial hexon sequence length of the PCR product. Phylogenetic trees were constructed using the neighbor-joining method and MEGA 11 with 1000 bootstrap values [30].

### 2.3. Statistical Analysis

Fisher’s exact test was used to analyze the significant differences among the groups. The significance level was set at *p* < 0.05. A 95% confidence interval (CI) was calculated for the estimates. IBM SPSS Statistics v 22 (IBM Corp., Armonk, NY, USA) was used for the statistical analyses.

## 3. Results

### 3.1. Prevalence of EAdV-1 DNA in the Nasal Swabs of Horses at the KRA

A total of 359 nasal swab samples were collected from horses reared on four KRA branch facilities in the Republic of Korea to investigate the prevalence of EAdV-1 DNA. EAdV-1 DNA was detected in 5 (1.4%) of the 359 horse nasal swab samples using a hexon-specific PCR (Table 1). One male horse (1/112, 0.9%), two geldings (2/123, 1.6%), and two female horses (2/124, 1.6%) were EAdV-1-positive (Table 1 and Table 2). The EAdV-1 positivity was 1.0% (1/96) in horses less than four years old, 1.1% (1/91) in four-year-old horses, and 1.7% (3/172) in horses over four years old. EAdV-1 was detected in the Seoul (2/178, 1.1%) and Jeju (3/71, 4.2%) facilities. Among the breeds, the Korean native pony showed the highest EAdV-1 infection rate (2.5%, 1/40), followed by the thoroughbred (1.4%, 4/292) and the mixed breeds (0%, 0/27). In the activity group, EAdV-1 positivity was 1.0% (3/288) in the racehorses, 3.0% (1/33) in the riding horses, and 2.6% (1/38) in the others. There were no statistically significant differences in EAdV-1 positivity between the groups (Table 1). As the relationship between EAdV-1 positivity and regional facilities was the lowest among the variables (*p* = 0.144), the EAdV-1 positivity between all facilities was combined and each facility was compared. The difference in EAdV-1 positivity in the Jeju facility approached a significance (*p* = 0.055), but not in the Busan (*p* = 0.591), Seoul, or Jangsu (*p* = 1) facilities.

Among the five EAdV-1-positive horses, one 11-year-old presented clinical respiratory signs and nasal discharge whereas the other four were clinically healthy (Table 2). No clinical signs were observed in any EAdV-1-negative horses. Using commercial real-time PCR kits with a QuantStudio 5 real-time PCR instrument (Applied Biosystems), the EAdV-1-positive samples were tested for other equine infectious respiratory agents, including equine influenza, EHV-1, EHV-4, and *Streptococcus equi* using Genesig Equine Flu H3N8 and H7N7 (Primerdesign, Camberley, UK), Genesig Equid Herpesvirus 1 (Primerdesign), Genesig Equid Herpesvirus 4 (Primerdesign), and Genesig Streptococcus equi (Primerdesign). None of the EAdV-1-positive samples showed co-infections with any of these agents.

### 3.2. Phylogenetic Analysis

**Figure 1:**
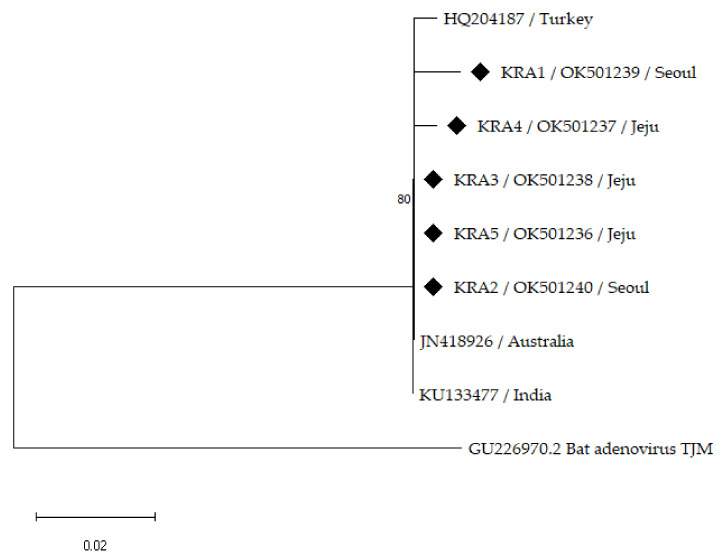
Phylogenetic tree constructed using neighbor-joining method based on partial hexon gene sequences of EAdV-1 with 1000 bootstrap replicates. Sequences of Korean horses are indicated by black diamonds with sample number, region of KRA facility, and GenBank accession number. GenBank accession numbers and country of foreign EAdV-1 strains are shown. Bat Adenovirus TJM (GU226970.2) was used for outgroup rooting.

Partial sequences of the hexon gene of five EAdV-1 isolates from the Korean horses were compared with the available foreign strains retrieved from GenBank, harboring 265 positions, to explore the genetic diversity of the EAdV-1 strains in the Republic of Korea (Figure 1). Previously reported foreign strains have been isolated from Australia, India, and Turkey. The partial nucleotide sequences of the five Korean EAdV-1-isolate hexon genes showed approximately 98.83–100% similarity amongst one another and 98.84–100% similarity with the foreign strains (Table 2). The sequences of the three Korean isolates (KRA2, −3, and −5) in this study were clustered with the retrieved isolate sequences from Australia and India. The Korean isolate (KRA4) was located separately from, but close to, the isolates from Turkey with 99.23% similarity whereas the Korean strain (KRA1) formed a separate branch, showing 98.83–99.23% similarity.

## 4. Discussion

Three hundred and fifty-nine nasal samples were collected and screened for EAdV-1 DNA to investigate the prevalence of EAdV-1 in horses reared at four KRA facilities in the Republic of Korea. EAdV-1 DNA was detected in 5 (1.4%) of the 359 horse nasal samples using an EAdV-1 hexon-specific PCR assay. Based on the detection of EAdV-1 DNA from the nasal swabs, the EAdV-1-positive rate in the Republic of Korea was low, which was consistent with previous studies reporting 2.9% in France [25], 3.9% in Belgium [26], and 1.4% in Turkey [22]. An EAdV-1 infection in horses is self-limiting and recovery occurs within 7–10 days [31,32]. EAdV-1-exposed horses do not normally retain recoverable viruses after 10 days [32]. As this study was conducted on horses reared at KRA facilities, most samples were collected from racehorses (289/359, 80.2%) examined by veterinarians on race days and certified as clinically healthy; only one horse showed nasal discharge as a respiratory symptom. A previous study using respiratory samples from healthy racehorses showed no EAdV-1 positivity (0/42, 0%) [24]. The relatively short recovery period after an EAdV-1 infection and nasal swab sample collection from mostly healthy horses at the KRA facilities could account for the low EAdV-1 positivity in this study.

The relevance of EAdV-1 hexon DNA was investigated on the basis of sex, age, region, breed, and activity (Table 1). No significant differences were observed between the groups in this study (*p* > 0.05). However, EAdV-1 positivity in the Jeju region was relatively high (4.2%, 3/71) and almost significant (*p* = 0.055) compared with the total regional positivity. Jeju Province is a key location in the horse industry in the Republic of Korea; in a survey conducted in 2020, there were 26,525 horses in the Republic of Korea and 14,759 were reared in Jeju Province (14,759/26,525, 55.6%) [33]. Additionally, 89.5% (3690/4124) of the breeding horses were reared in Jeju Province [33], although Jeju Province is only 1.8% of the area of the Republic of Korea (1850/100,412 km^2^) [34]. EAdV-1 infections infrequently occur in immunocompetent foals, but have been correlated with pneumonia irrespective of the breed [7]. Therefore, although the prevalence of EAdV-1 is low in the Republic of Korea, evaluating EAdV-1 in respiratory infection candidates would be beneficial to prevent possible EAdV-1 outbreaks in horses in the Jeju region, considering the high horse population density and potential risk to foals.

Partial sequences of the EAdV-1 hexon gene strains were used to establish phylogenetic relationships among the Korean isolates and foreign strains obtained from GenBank (Figure 1). The Korean EAdV-1 strains showed a low genetic diversity among themselves (98.83–100% similarity), but a slightly greater diversity than the foreign strains (98.84–100% similarity), consistent with a previous study [22]. An isolate (HQ204187) from Turkey showed a low genetic diversity (0.4%) of the partial EAdV-1 hexon gene compared with an Australian strain (JN418926) [22]. In a phylogenetic analysis, one isolate (KRA5) from an EAdV-1-positive horse with respiratory symptoms was closely clustered with clinically normal Korean isolates without clinical signs as well as the Australian and Indian strains. The Australian strain (JN418926) was isolated from a clinically normal foal in 1972 [27] and the Indian strain (KU133477) was isolated from a healthy horse in 2011. The Turkish strain (HQ204187) was isolated from a horse with respiratory symptoms in 2012 [22]. Additionally, 15 EAdV-1 partial hexon genes detected in horses in the United States in 2006 were identical to the Australian strain, including 2 isolates from foals with respiratory signs and 13 isolates from clinically healthy foals and horses [24], suggesting that the EAdV-1 partial hexon gene has a low degree of genetic diversity regardless of the clinical signs.

A limitation to this study was that only three reference strains from Australia, India, and Turkey were recruited for the phylogenetic analysis. Molecular detection using an EAdV-1 partial hexon-specific PCR assay [23] was reported in Belgium [26], France [25], and the United States [24]; however, no EAdV-1 isolate nucleotide sequences have been released or deposited in GenBank (accessed on 11 March 2022). One partial nucleotide sequence of the EAdV-1 hexon gene of the Turkish strain and two whole EAdV-1 strain nucleotide sequences from Australia and India are available. Thus, further phylogenetic studies with various EAdV-1 nucleotide sequences are required for a better presentation of the data.

Although an EAdV-1 infection induces subclinical or mild clinical signs in most horses [3], mixed infections with other viruses and bacteria can cause more severe upper respiratory diseases [31,32,35]. EHV-1, EHV-4, EIV, and *Streptococcus equi* are the most common pathogens causing infectious upper respiratory tract equine diseases [36]. Therefore, multiple infections with these pathogens were tested for in the EAdV-1-positive horses. However, no co-infection with these important equine respiratory pathogens was identified in the EAdV-1-positive horses in this study. The EAdV-1-positive horse with clinical signs recovered spontaneously within 5 days without complications and the neighboring horses showed no clinical signs. The mild respiratory signs in the EAdV-1-positive horse were thought to be caused by a solitary EAdV-1 infection or unidentified pathogens other than the above-mentioned common equine respiratory pathogens. Most EAdV-1-positive horses (4/5, 80%) in this study were asymptomatic, possibly due to the self-limiting nature of EAdV-1 and a solitary EAdV-1 infection. However, a further study on the virulence of EAdV-1 infections in horses would be required.

## 5. Conclusions

This study provided information on the molecular prevalence of EAdV-1 in nasal swab samples from horses reared at KRA facilities in the Republic of Korea. To the best of our knowledge, this is the first report of an EAdV-1 infection in horses from the Republic of Korea. The prevalence of EAdV-1 was low (1.4%); however, EAdV-1 positivity in the Jeju region was relatively high (4.2%). Considering the importance of Jeju Province as a key location in the horse industry in the Republic of Korea, EAdV-1 could be a potential equine respiratory disease cause in the Jeju region. Although the *p*-value for the Juju region was 0.055 and only approached a significance, it could be helpful to include EAdV-1 in equine respiratory disease tests. The identified EAdV-1 strains exhibited a low genetic diversity (98.8–100% nucleotide similarity) and three out of the five Korean strains were closely related to the foreign strains. Considering the limited availability of reported EAdV-1 sequences, further studies with more EAdV-1 sequences from various countries could be beneficial for analyzing the global phylogeny of EAdV-1.

## Figures and Tables

**Table 1:** Prevalence of EAdV-1 detected using a PCR on nasal swabs from horses at KRA facilities in the Republic of Korea in 2021.

Group	No. Tested	EAdV-1		
		No. Positive (%)	95% CI	*p*-Value
Sex				
Male	112	1 (0.9)	0–0.03	1
Gelding	123	2 (1.6)	0–0.04	
Female	124	2 (1.6)	0–0.04	
Age				
<4 years	96	1 (1.0)	0–0.03	1
=4 years	91	1 (1.1)	0–0.03	
>4 years	172	3 (1.7)	0–0.03	
Region				
Seoul	178	2 (1.1)	0–0.03	0.144
Busan	80	0		
Jangsu	30	0		
Jeju	71	3 (4.2)	0–0.09	
Breed				
Thoroughbred	292	4 (1.4)	0–0.03	0.646
Korean native pony	40	1 (2.5)	0–0.08	
Mixed	27	0		
Activity				
Race	288	3 (1.0)	0–0.02	0.258
Riding	33	1 (3.0)	0–0.09	
Others	38	1 (2.6)	0–0.08	
Total	359	5 (1.4)	0–0.03	

No.: number of horses; CI: confidence interval.

**Table 2:** Details of PCR-tested EAdV-1-positive horses and amplicon accession numbers.

Sample	Region	Sex	Age	Clinical Signs	Accession Number	Activity
KRA1	Seoul	Female	5	None	OK501239	Race
KRA2	Seoul	Male	4	None	OK501240	Race
KRA3	Jeju	Gelding	5	None	OK501238	Race
KRA4	Jeju	Gelding	3	None	OK501237	Riding
KRA5	Jeju	Female	11	Nasal discharge	OK501236	Others

## Data Availability

Sequences identified in this study were deposited in the GenBank database under the accession number OK501236-OK501240.
